# COVID-19-Associated Parotitis in a 10-Week-Old Male

**DOI:** 10.7759/cureus.31054

**Published:** 2022-11-03

**Authors:** Robin Brehm, Lakshmi Narayanam, Grace Chon

**Affiliations:** 1 Pediatrics, Nemours Children's Health System, Philadelphia, USA; 2 Pediatrics, Sidney Kimmel Medical College, Philadelphia, USA

**Keywords:** coronavirus disease 2019, covid-19, sars-cov-2 parotitis, sars-cov-2, sars-cov-2 in pediatric patients, viral parotitis, neonatal covid-19 infection, neonatal parotitis, covid-19 retro

## Abstract

We describe a case of parotitis associated with coronavirus disease 2019 (COVID-19) in a young male infant. His presenting symptom at the time of diagnosis of COVID-19 was unilateral facial swelling. He then developed upper respiratory infection symptoms and proceeded to recover over a period of about a month. Testing for other causes of parotitis was unrevealing. Other cases of COVID-19-associated parotitis have been presented in the literature, but this case is by far the youngest child noted, and is a useful reminder to pediatricians and general practitioners to consider COVID-19 as a cause of parotitis. Additionally, it sheds light on possible transmission and pathophysiology of COVID-19 in the salivary glands, as several other authors have noted.

## Introduction

Over the past two years, coronavirus disease 2019 (COVID-19), a disease secondary to the SARS-CoV-2 virus, has transformed from a novel, emerging pathogen, to an ever-present consideration in patient care. COVID-19 infection can present a wide range of clinical presentations, from asymptomatic to mild upper respiratory infection, to fulminant respiratory failure due to pneumonia [1]. It can affect many body systems. In this case report, we highlight a more unusual presentation of this highly prevalent disease in a young infant with parotitis.

## Case presentation

A 10-week-old male presented to the outpatient pediatric office for evaluation of “facial swelling.” The swelling started the night prior, and it had neither improved nor worsened since it was first noted. At the time of initial presentation, the patient was without fever or upper respiratory infection symptoms. Compared to his baseline, there was a decrease in appetite, but he was still feeding normally and producing normal urine output. On exam, he had unilateral, right-sided facial swelling with a firm, tender mass consistent with an inflamed parotid gland. No purulent drainage was appreciable from Stensen’s duct. His examination was otherwise normal.

His most recent well-check was nine days prior. At that visit, he was noted to have appropriate growth parameters and received his two-month vaccinations. He was born via a healthy term delivery and received all appropriate screening tests after birth. On review of his birth records, his mother was up to date on her vaccinations, including measles, mumps, and rubella (MMR), and tested negative for HIV during pregnancy. His past medical history was notable for a brief resolved unexplained event (BRUE), for which he was seen at a pediatric emergency department when 19 days old.

As he presented to care during the initial Omicron COVID-19 surge, a rapid SARS-CoV-2 polymerase chain reaction (PCR) test was ordered and was found to be positive on initial presentation. The exam and history were most consistent with viral parotitis. While bacterial parotitis is a consideration in this age group, he was well-appearing, afebrile, and taking fluids. The family was eager to avoid emergency room care during a COVID-19 surge with an infant, and a plan was made for close pediatric follow-up with their primary care doctor. The family was given anticipatory guidance regarding symptoms, including a plan to go to the emergency room if he became febrile, had decreased oral intake, or decreased urine output.

Over the next few weeks, his parents were in close contact with their pediatrician to monitor his illness, and his symptoms initially seemed to wane with supportive care. He subsequently developed some symptoms of rhinorrhea and cough but recovered from these as well. However, 21 days after the initial encounter, his parents notified their pediatrician that his facial swelling seemed to be worsening again. They continued to deny fever but reported increasing discomfort at being touched on the right cheek. On exam, he had tenderness and fullness of the right cheek and was still without purulent discharge from Stensen’s duct. He displayed no other symptoms characteristic of COVID-19 at this encounter.

At this time, a more complete workup was undertaken for further evaluation. An ultrasound was ordered but was not obtained by the parents due to insurance barriers. He was found to have leukocytosis with a white blood cell count of 22.1 µL (0.2 x 109), hemoglobin of 10.6 g/dL (106 g/L) (appropriate for age, physiologic nadir), hematocrit at 34.2%, platelets elevated at 745/µL (7.5 x 109), and mean corpuscular volume (MCV) of 75 µm3 (75 fL). His differential was notable for 28% neutrophils, 59% lymphocytes, 8% monocytes, 2% eosinophils, 1% basophils, and 2% activated lymphocytes. Cytomegalovirus (CMV) and Epstein-Barr virus (EBV) titers were drawn, and both were noted to be IgG positive, and IgM negative. Mumps was IgG negative and IgM negative. Given leukocytosis and initial improvement followed by clinical worsening, there was a concern for possible superinfection, and a seven-day course of amoxicillin/clavulanic acid was prescribed.

The patient was seen for a follow-up nine days following the course of antibiotics and was noted to be substantially improved. His fullness over the right parotid gland resolved, and no longer seemed tender to palpation.

## Discussion

We interpret our patient’s clinical data and course as most consistent with viral parotitis secondary to COVID-19. The subsequent worsening may potentially be bacterial superinfection, which is resolved with antibiotics, but it also has been noted that COVID-19-related parotitis can take over a month to resolve [2]. Several case reports of parotitis secondary to COVID-19 have been noted [2-8], but this case is remarkable as the patient was noted to be the youngest to have COVID-19-related parotitis. The previous youngest patient was noted by Ekemen et al. and was aged four years [4]. One must be cautious in a patient of this age, as bacterial parotitis is more common in children under three months. The chief distinguishing factor is the absence of purulent discharge noted from Stensen’s duct on an exam, which is a common feature of parotitis due to bacterial infection.

When there is parotid swelling and purulent discharge from the Stensen's duct, neonatal bacterial parotitis should be considered as a diagnosis. The presentation may be variable as the patients are only febrile 50% of the time and have other vague symptoms like irritability and excessive crying. About 10-20% of cases have shown bilateral parotid swelling [9]. To diagnose parotitis, a bacterial culture of the discharge is required, most commonly showing *Staphylococcus aureus* [9-14]. There is an incidence of one per 100,000 in full-term babies and an incidence of 3.8 to 14 per 10,000 neonatal intensive care unit (NICU) babies [10]. In addition to pre-term birth, other risk factors for neonatal parotitis include low birth weight, male gender, prolonged nasogastric feeding, immunosuppression, contaminated feeds, and dehydration [10-12]. The most common treatment is antibiotics such as cephalosporins [9,14]. In a minority of cases, surgical drainage might be necessary [12].

A review of other case reports suggests that our child’s presentation is, in fact, most consistent with parotitis secondary to COVID-19 [2-8]. Most cases of parotitis-like symptoms present unilaterally. While a blood count was not performed at the first encounter, several other patients demonstrated initial leukopenia. Our patient had leukocytosis on the second encounter, which may be attributable to a superimposed bacterial infection. A chief distinguishing feature of the patients included in these case reports is that no patient demonstrated purulent discharge following parotid gland massage. There was a varied distribution of COVID-19 symptoms, ranging from asymptomatic to hypoxia requiring hospitalization. Common imaging findings among patients who tested positive for COVID-19 and exhibited symptoms of acute parotitis included diffuse enlargement of the parotid gland, peri-parotid fat stranding, and fluid tracking into surrounding musculature. The resolution of parotitis-like symptoms varied from three to 50 days from symptom onset [2,4]. Most patients diagnosed with acute parotitis were either asymptomatic of typical COVID-19 infection or demonstrated COVID-19 symptoms prior to the development of parotitis [2-5,7,8], but one patient was noted to have parotitis as his initial symptom [6]. Most patients seem to have a resolution of their symptoms with supportive care, suggesting this is an adequate initial treatment strategy. However, consideration of secondary infection is reasonable, as our patient had a biphasic illness and improved with subsequent antibiotic treatment.

While COVID-19 is thought to be largely transmitted via droplets, an animal model in macaques with a related severe acute respiratory syndrome coronavirus (SARS-CoV) virus suggests that direct viral infection of the parotid gland is possible through angiotensin-converting enzyme 2 (ACE2) receptors [15]. ACE2 and transmembrane protease serine 2 (TMPRSS) expression was confirmed in the salivary glands of COVID-19-positive patients, suggesting that the parotid gland is a direct site of COVID-19 infection [16]. Additionally, To et al. noted the shedding of the SARS-CoV2 virus in the saliva of a small series of patients [17]. Other authors suggest that immune dysregulation and inflammation post-COVID-19 could also cause parotitis-like symptoms [3].

## Conclusions

Given the high prevalence of COVID-19 in our community, and our attempts as physicians to quell the spread of the disease, a COVID-19 test is an important consideration for physicians when faced with any patient with parotitis. Our patient's first symptom of COVID-19 was parotitis, which leads to the conclusion that providers should consider COVID-19 in the differential diagnosis even in a patient without other symptoms. Parotitis may be a more common manifestation of COVID-19 than has been previously noted. As there are now several antiviral treatment options available for older children and adults, prompt consideration of COVID-19 would allow treatment with antiviral therapies without delay. One must be vigilant of unusual manifestations of this now-common illness.
